# Supplementary material for: Effects of velocity based training vs. traditional 1RM percentage-based training on improving strength, jump, linear sprint and change of direction speed performance: A Systematic review with meta-analysis
Source: PLoS One. 2021 Nov 18;16(11):e0259790. doi: 10.1371/journal.pone.0259790 (PMC8601436; doi:10.1371/journal.pone.0259790)
Supplement: S1 Table — (DOCX) [file pone.0259790.s002.docx]

Supplemental Table 1. Searching strategy and syntax

| Term | Key words |
| --- | --- |
| 1. terms related with Velocity based training | “Velocity based training”, VBT, velocity, speed, resistance |
| 1. terms related with Percentage based training | “Strength training”, “power training”, “resistance training”, “percentage based training”; |
| 1. Terms related with performance | Strength, power, performance, “change of direction”, agility, endurance, speed, sprint |
| Searching syntax in PubMed | ((((velocity based training[Text Word]) OR (vbt[Text Word])) OR ((velocity[Text Word] OR speed)[Text Word] AND ( strength[Text Word] OR resistance[Text Word] OR power[Text Word] OR force)[Text Word])) AND ((strength training"[Text Word]) OR (resistance training[Text Word]) OR (power training[Text Word]) OR (percentage based training[Text Word])) AND ((strength[Text Word]) OR (power[Text Word]) OR (performance[Text Word]) OR (change of direction[Text Word]) OR (agility[Text Word]) OR (endurance[Text Word]) OR (speed[Text Word]) OR (sprint[Text Word]))) |
| Searching syntax in Web of Science | ("Velocity based training"  OR vbt or ((velocity or speed) and (strength or resistance or power))) AND  SU: ("Strength training" or "power training" or "resistance training" or "percentage based training") AND SU: (Strength or power or performance or "change of direction" or agility or endurance or speed or sprint) |
| Searching syntax in CNKI | (SU=“基于速度的力量训练” OR SU=“速度负荷”) AND (SU=力量 OR SU=抗阻) |
