# Supplementary material for: Effects of velocity based training vs. traditional 1RM percentage-based training on improving strength, jump, linear sprint and change of direction speed performance: A Systematic review with meta-analysis
Source: PLoS One. 2021 Nov 18;16(11):e0259790. doi: 10.1371/journal.pone.0259790 (PMC8601436; doi:10.1371/journal.pone.0259790)
Supplement: S2 Table — (DOCX) [file pone.0259790.s003.docx]

Supplemental Table 2. Modified risk of bias assessment scale (23, 24)

| **No** | **Items** | **Scores** |
| --- | --- | --- |
| 1 | Clear inclusion criteria | 0-2 |
| 2 | Clear description of the participants training experience | 0-2 |
| 3 | Random allocation of the participants to groups | 0-2 |
| 4 | Clearly defined intervention | 0-2 |
| 5 | Similarity test at baseline for all groups | 0-2 |
| 6 | Use of a control group that did not perform resistance training | 0-2 |
| 7 | Clearly defined outcome variables | 0-2 |
| 8 | Adequate familiarization period | 0-2 |
| 9 | Appropriate between-group statistical analysis | 0-2 |
| 10 | Point measures of variability | 0-2 |
|  | Total | 0-20 |
