# Supplementary material for: Effects of velocity based training vs. traditional 1RM percentage-based training on improving strength, jump, linear sprint and change of direction speed performance: A Systematic review with meta-analysis
Source: PLoS One. 2021 Nov 18;16(11):e0259790. doi: 10.1371/journal.pone.0259790 (PMC8601436; doi:10.1371/journal.pone.0259790)
Supplement: Attachment — Submitted filename: Response to Reviewers-0815.docx [file pone.0259790.s005.docx]

Dear Professor Daniel Boullosa

Many thanks for your letter and the comments from the reviewers regarding our paper.

After carefully checking the manuscript, we have revised it according to the reviewer’s comments. We submit here the modified version as well as a list of change.

If you have any question, please let me know.

Thank you again.

Kind regards

**Response to editor’s comments:**

Thanks for your great comments on our paper. We have revised our paper according to your comments and make sure that the manuscript will be up to publishable level with your help.

Abstract:
1. I would change “linear speed” to “linear sprint”. Linear speed is already performance. It would be like jump height. Therefore, it would be better to use: linear sprint throughout the manuscript.

Response: We agree with your suggestions, and have already changed “linear speed” into “linear sprint” throughout the manuscript. Thank you.

2. Abstract. Methods. SD has not been previously defined.

Response: We have now defined SD. Thank you.

3. Abstract. Results. Why sometimes you show MD and others SMD? I would use only SMD. Please see my specific comments below.

Response: From a practical standpoint, we prefer MD in current study. This is because MD is simpler and more meaningful in practice. For example, if we tell the coaches that VBT can increase 1RM of squat by 10kg in 4 weeks, it will be easy to be understood. On the other side, if we tell them that 1RM can improve by 0.4 whole standard deviation it is very confusing. As a support, the American Psychological Association (APA) Task Force on statistical inference also suggests that if the units of measurement are meaningful on a practical level, then a MD is superior to a SMD. Thus, when all studies report the outcome using the same scale or the same tests, we used MD, otherwise we used SMD. that’s the reason why we sometimes show MD and others SMD. Thank you again.

Introduction
5. Third paragraph. These research to date… should read: these researches…

Response: We have corrected that. Thank you.

6. Therefore, A systematic review with… should read: Therefore, a systematic…

Response: We have corrected that too. Thank you.

Methods
Search strategy.

7. syntax was shown in supplemental table 1… should read: syntax are shown…

Response: This has been corrected.

8. Risk of bias assessment within individual studies

Two authors (The second… should read: the second

Response: We have corrected that. Thank you.

9. Brughelli et al [23]…should read: al.

Response: This has been corrected. Thank you.

Statistical analysis

10.Since MD can be highly influenced by sample characteristics. Would not it be better using always SMD? SMD can amend some of the problems shown by MD, since it includes SD in the calculations.

Response: Thank you for your comment. Previously (response to comment 3) we have justified our approach. Thank you.

11. Both algorithms of MD and SMD in speed and COD outcomes was adjusted…should read: were

Response: This has been corrected. Thank you.

Results
Search results

12. At last, 6 studies included in the qualitative…should read: were included

Response: This has been corrected. Thank you.

Risk of bias assessment

13. All of studies got high scores… should read: All studies or all the studies

Response: This has been corrected. Thank you.

Studies’ Characteristics

14. characteristics of 6 studies included in qualitative analysis was…should read: were

Response: This has been corrected. Thank you.

15. Among which, Only 1 study…should read: only

Response: This has been corrected. Thank you.

Quantitative analysis.

16. load velocity with 40%1RM. Shouldn’t be 60% 1RM?

Response: Thank you for this comment too. We have corrected that.

Discussion
17. IMHO, I miss you highlight the methodological differences between both approaches throughout the Discussion, especially in the Practical Applications section. Thus, you may help readers to better choose the proper method.

Response: We have added the methodological differences between VBT and PBT in the discussion and practical applications section following your suggestions. Thank you .

Strength performance

18. improvement in musclular strength…should read: muscular

Response: This has been corrected. Thank you.

19. similarities of biomechenics to a various…should read: biomechanics

Response: We corrected that. Thank you.

20. PBT ( ES = 1.25) With the…should read: with

Response: This has been corrected. Thank you.

21. Please rewrite this sentence: This likely accounted for subtle decreases in load adjusted in accordance with targeted velocity.

Response: We have rewritten this sentence. Thank you.

22. velocity testing device. although the…should read: device, although

Response: We corrected that too. Thank you.

23. …. and placebo effect from the velocity testing device. although the research conducted by Chen et al. and Held et. al. did not provide the velocity data of training repetitions [20,29], it could be inferred that participants might perform even higher repetition velocity due to enhancing motivation and competitiveness from feedback of velocity[12]. Please split or rewrite this sentence.

Response: We have rewritten this sentences.

24. Furthermore, Interestingly,…should read: interestingly

Response: We have corrected that. Thank you.

25. and 48 hours in VBT was superior…should read: were

Response: This has been corrected. Thank you.

26. VBT should produced a better…should read: would produce

Response: This has been corrected. Thank you.

27. while it was likely for PBT to…should read: it is likely

Response: This has been corrected. Thank you.

28. With respect to the effects in favor of VBT. This sentence should go on.

Response: After considerable thinking, we decided to delete this incompletely sentence.

29. more repetition…should read: more repetitions

Response: This has been corrected. Thank you.

30. at maiximum intended velocity…should read: maximum

Response: This has been corrected. Thank you.

31. threshold type Ⅱ fiber which have a greater relative hypertrophy than type Ⅱ fiber…should be: type II and type I, respectively

Response: This has been corrected. Thank you.

32. for above metioned factors…should read: mentioned

Response: This has been corrected. Thank you.

33. device[44]. this finding…should read: This

Response: This has been corrected. Thank you.

34. Considering the methodology involved the use of post minus pre intervention value in our meta-analysis, it could. Please use comma when necessary

Response: We have used comma. Thank you.

4.2 Jump performance

35. and muslce fiber type composition …should read: muscle

Response: This has been corrected. Thank you.

36. development [45] and muscle-tendo …should read: muscle-tendon.

Response: This has been corrected. Thank you.

37. Furthermore, Banyard et al. reported that the mean deviation of
sessional repetition velocity was greater for the PBT (-13.6 ± 6.8%) contrasted to VBT(-0.2 ± 5.2%) [22]. You should explain why these authors reported this and how can influence jump height adaptations.

Response: We added a sentence to explain that. Thank you for your comment.

38. is also evdence: evidence

Response: This has been corrected. Thank you.

39. intervetion exercise: intervention

Response: This has been corrected. Thank you.

40. resistance training [47], The higher movement… should read: the

Response: This has been corrected. Thank you.

Linear Speed and CODs performance

41. Evidence showed that the squat strength and sprint performance existed very large significant correlation ( r= -0.77; p = 0.001) [48]. This sentence is hard to follow.

Response: We have rewritten this sentence. Thank you.

42. above metioned: mentioned.

Response: This has been corrected. Thank you.

43. may also impaire resistance: impair.

Response: This has been corrected. Thank you.

44. in both group… should read: in both groups [18].

Response: This has been corrected. Thank you.

45. This negetive effects may: negative.

Response: This has been corrected. Thank you.

46. This negligible results: these.

Response: This has been corrected. Thank you.

47. still needed to distingush the difference: distinguish.

Response: This has been corrected. Thank you.

48. CODs (ES = 0.67-0.79) favorited VBT compared to PBT [22]: favored.

Response: This has been corrected. Thank you

49. for this results: these

Response: This has been corrected. Thank you.

50. may be the same as above metioned such: mentioned

Response: This has been corrected. Thank you.

51. recovery. this implies that pactitioners are… shoud read: This implies that practitioners…

Response: We corrected that too. Thank you.

4.4 Limitations

52. all results was graded: were

Response: We corrected that. Thank you.

Conclusion
53. less excaustive nature: exhaustive

Response: This has been corrected. Thank you.

TABLES
Table 2.
54. Song Chen 1995. I cannot access to this reference. But did they use as a reference load? the maximal power load? or all of them trained at the same velocity? It is strange that they compared PBT and VBT in 1997 when the first publications about using VBT instead of PBT are from 2010.
I guess they compared power-based training, but this is not the same idea, since the use power, not velocity, for monitoring and programing the training load.

Response: After carefully rechecking this reference we have made sure that this study by Song Chen 1995 trained the VBT group at the same velocity by adjusting the load with a self-made velocity testing devices, which is in accordance with our definition of VBT described in the method. As you correctly mentioned, Chen’s concept was associated with power-based training at that time. However, the author puts forward some quite new and unique insight on velocity load such as the range of speed control (velocity loss), increasing the speed with controlled weight, and increasing weight with controlled speed. This same author also published several articles from his doctoral thesis to state the concept of velocity load training and provided suggestions on how to prescribe the load using velocity. Please find below one of his articles’ abstract published in 1994. The full reference for that is Song Chen, Qiwei Ma. Training theory and method of developing velocity-force by quantitative control of movement speed. Journal of Beijing University of Physical Education. 1994; 17(2):78-89.

55. Some of the abbreviations described in the footnote are not used in the table.

Response: Thank you for this suggestion too. After checking we have deleted the extra abbreviations.

Table 3.

56. I miss some of the included studies here

Response: You are right, two studies were not in the table 3. Because table 3 is to show the results of dealing with the multiplicity in included studies. Therefore, we did not include the study without multiplicity in table 3. Thank you.

FIGURES
57. Figure 3A. I guess this should be Song;, C. and M. qiwei, Effects of fixed velocity training methods on load velocity profile. Journal of Beijing University of Physical Education, 1995. 18(3):p. 81-88.

Response: Yes, that’s Song Chen and Qiwei MA. We have corrected it in the figure. Thank you.

58. Figure 3D. how have you calculated the weight in Fig. 3D? It is weird that a study with 10 subjects means the 1.9% and other with 12 the 72.4% and the third one with 15 subjects and only the 25.7% of the total weight.

Response: Thank you for this point too. This is because we adopted the random-effects model to calculate the MD in figure 3D via Revman software. Weighting within the random-effects model assumes two sources of variability in effects, one from sampling error and one from study level differences. However the latter is based on the variability in effect sizes across the group of studies and it has been taken to imply that the random-effects model assumes a distribution of true population effects from which the observed studies are sampled. The reason for this large difference in weight distribution may be due to the fact the third one with much bigger SD than the other two studies results in large study level difference.
